# Targeting of the Eukaryotic Translation Initiation Factor 4A Against Breast Cancer Stemness

**DOI:** 10.3389/fonc.2019.01311

**Published:** 2019-12-06

**Authors:** Sangita Sridharan, Megan Robeson, Diwakar Bastihalli-Tukaramrao, Cory M. Howard, Boopathi Subramaniyan, Augustus M. C. Tilley, Amit K. Tiwari, Dayanidhi Raman

**Affiliations:** ^1^Department of Cancer Biology, University of Toledo Health Science Campus, Toledo, OH, United States; ^2^Department of Pharmacology & Experimental Therapeutics, University of Toledo Health Science Campus, Toledo, OH, United States

**Keywords:** breast cancer stemness, eIF4A, Rocaglamide A, chemoresistance, actionable targets, cell death, ABC transporters, Triple-negative breast cancer (TNBC)

## Abstract

Breast cancer stem cells (BCSCs) are intrinsically chemoresistant and capable of self-renewal. Following chemotherapy, patients can develop minimal residual disease due to BCSCs which can repopulate into a relapsed tumor. Therefore, it is imperative to co-target BCSCs along with the bulk tumor cells to achieve therapeutic success and prevent recurrence. So, it is vital to identify actionable molecular targets against both BCSCs and bulk tumor cells. Previous findings from our lab and others have demonstrated that inhibition of the emerging drug target eIF4A with Rocaglamide A (RocA) was efficacious against triple-negative breast cancer cells (TNBC). RocA specifically targets the pool of eIF4A bound to the oncogenic mRNAs that requires its helicase activity for their translation. This property enables specific targeting of tumor cells. The efficacy of RocA against BCSCs is unknown. In this study, we postulated that eIF4A could be a vulnerable node in BCSCs. In order to test this, we generated a paclitaxel-resistant TNBC cell line which demonstrated an elevated level of eIF4A along with increased levels of cancer stemness markers (ALDH activity and CD44), pluripotency transcription factors (SOX2, OCT4, and NANOG) and drug transporters (ABCB1, ABCG2, and ABCC1). Furthermore, genetic ablation of eIF4A resulted in reduced expression of ALDH1A1, pluripotency transcription factors and drug transporters. This pointed out that eIF4A is likely associated with selected set of proteins that are critical to BCSCs, and hence targeting eIF4A may eliminate BCSCs. Therefore, we isolated BCSCs from two TNBC cell lines: MDA-Bone-Un and SUM-159PT. Following RocA treatment, the self-renewal ability of the BCSCs was significantly reduced as determined by the efficiency of the formation of primary and secondary mammospheres. This was accompanied by a reduction in the levels of NANOG, OCT4, and drug transporters. Exposure to RocA also induced cell death of the BCSCs as evaluated by DRAQ7 and cell viability assays. RocA treatment induced apoptosis with increased levels of cleaved caspase-3. Overall, we identified that RocA is effective in targeting BCSCs, and eIF4A is an actionable molecular target in both BCSCs and bulk tumor cells. Therefore, anti-eIF4A inhibitors could potentially be combined synergistically with existing chemo-, radio- and/or immunotherapies.

## Introduction

Among the cancer fatalities in women, breast cancer (BC) ranks as a second leading cause of death. According to the 2019 estimates, the morbidity and mortality for BC in women stands at 30 and 15%, respectively (1). The metastasis of BC to the lungs, bone, and brain is the main precipitating cause of lethality. The inter- and intra-tumor clonal heterogeneity and plasticity of tumor cells observed in triple-negative BC (TNBC) form the leading cause of chemoresistance, tumor relapse, and poor prognosis (2–8). A small subset of tumor cells residing in the tumor called as BC stem cells (BCSCs) or tumor initiating cells are attributed to such clinically resistant cases of BC.

Cancer stem cells (CSCs) were identified as a “side population” (SP) by flow cytometric analyses based on the efflux of Hoechst dye by the family of adenosine triphosphate (ATP)-binding cassette (ABC) drug transporters such as ABCB1 and ABCG2 present at the plasma membrane (9). This perhaps is one of the mechanisms by which CSCs bypass chemotherapy through efflux of xenobiotics (including anti-cancer drugs) to the exterior of the cell leading to their survival in patients. Interestingly, SP cells were found to be significantly enriched in ER- and TNBC patient biopsies (9). BCSCs are generally characterized by increased intracellular aldehyde dehydrogenase (ALDH) activity and/or the transmembrane glycoprotein called as cluster of differentiation 44 (CD44) (10). ALDHs are a set of detoxification isoenzymes implicated in retinoid metabolism. Retinoid-mediated signaling plays an important role in embryonic stem cells (11) and detoxification of drugs in a cancer setting (12). CD44 is generally involved in cell-cell and cell-matrix adhesions as well as cell migration (13, 14). A subset of BCSCs co-expresses both CD44 and ALDH markers and these BCSCs are considered highly aggressive and metastatic (10, 15, 16). BCSCs usually express a combination of pluripotency transcription factors such as SOX2, OCT4, and NANOG. In TNBC, SOX2 promotes proliferation and metastasis (17). An increased expression of NANOG serves as a prognostic indicator and suggested to be co-expressed with the CD133 marker (prominin1) (18–21). In surgical TNBC patients, OCT4 has been shown to predict poor patient outcome (22). Expression of SOX2, NANOG, and OCT4 transcription factors correlated with poor differentiation, advanced BC stage and worst survival in BC patients (23).

The expression of cell surface and subcellular markers of BCSCs adapt in response to the alterations in the tumor microenvironment (TME). Mesenchymal and epithelial phenotypes of BCSCs have been reported to display differential gene expression profiles which may contribute to heterogeneity and differential chemoresistance (24). Interconversion between the two BCSC phenotypes occurs at a slow rate (24, 25). BCSCs can also bi-directionally interconvert between bulk tumor cell and stemness states based on temporal and spatial cues in the microenvironment of the BCSCs (21). This creates a remarkable genetic and/or epigenetic heterogeneity and cellular plasticity in BCSCs and bulk tumor cell pools which presents a clinical challenge. The ability of BCSCs to self-renew, differentiate into bulk tumor cells and resist radio- and chemotherapy allows them to remain viable following therapy constituting the minimal residual disease (MRD). Subsequently, the BCSCs can differentiate and repopulate the whole tumor leading to relapse. Therapy failure after multiple rounds of exposure to the chemotherapeutic agents will lead to aggressive tumor behavior resulting in distant metastases or metastasis of the metastases that culminates in mortality.

TNBC patients often exhibit the paradox of an initial response followed by refractoriness to neoadjuvant chemotherapy (26, 27). This is especially true in taxane therapy with docetaxel wherein there is a therapy response initially followed by development of resistance (28). Therefore, there is an unmet need for novel and specific therapies to overcome the chemoresistance possibly arising from BCSCs, i.e., develop BCSC-directed therapies. Also, considering the interconversions between the bulk tumor cells and BCSCs it is necessary to co-target BCSCs along with bulk tumor cells in order to achieve clinical success and most importantly improve the longevity in patients with metastatic BC (29–33).

In our drug screen for developing BCSC-directed therapy, we found that Rocaglamide A (RocA), a flavagline compound that targets the eukaryotic translation initiation factor 4A1 (eIF4A1), is efficacious against BCSCs. eIF4A1 (will be referred to as eIF4A throughout) is a vital component of the eukaryotic translation initiation eIF4F complex that facilitates translation of many oncogenic proteins. eIF4A, being an mRNA helicase, unwinds key stem-loop-structured (SLS), oncogenic mRNAs such as baculoviral IAP repeat containing 5 (BIRC5) or survivin (survival), MDM2 (antagonizes p53), MCL1 and BCL2 (anti-apoptotic factors), Rho kinase1 (ROCK1, cell migration), SIN1 (part of mTORC2 complex, cell migration), Mucin1-C (MUC1-C), Cyclin D1 and D3 (proliferation) among others for efficient ribosome scanning and their translation. Overall, these proteins are implicated in survival and metastasis of BCSCs and bulk tumor cells. Our earlier (34) and current findings suggest that RocA may be a useful compound to target both BCSCs and bulk tumor cells.

## Methods

### Cell Culture

The human triple-negative breast cancer cell lines: MDA-MB-231, lung trophic MDA-MB-231-LM2-4175(represented as MDA-MB-4175), bone-trophic MDA-MB-231-BoM-1833 [represented as MDA-MB-1833 (35)], MDA-Bone-Un (MDA-MB-231 cells re-isolated from mouse bone metastatic lesions) (36, 37) and SUM-159PT (38) were routinely maintained in Dulbecco's modified eagle medium (DMEM) (GE Healthcare Life Sciences, Pittsburgh, PA, Cat. #—SH30243.01) supplemented with 4 mM L-glutamine, 4.5 g/L glucose, sodium pyruvate, 10% heat-inactivated fetal bovine serum (FBS) (Denville Scientific, Swedesboro, NJ, Cat. #—FB5001-H), and 1% Penicillin (100 I.U.)/Streptomycin (100 μg/ml) (Corning, Corning, NY, Cat.#−30-002-CI) at 37°C in a humidified incubator containing 5% CO_2_.

### Isolation of BCSCs Based on Aldehyde Dehydrogenase (ALDH) Activity

MDA-Bone-Un and SUM-159PT tumor cells, MDA-Bone-Un eIF4A CRISPR control (CC) and knockout cells (KO) cultured in a monolayer were trypsinized and BCSCs with high ALDH activity were isolated by employing the ALDEFLUOR™ Kit (Stem Cell Technologies, Vancouver, BC, Canada, Cat. #−01700) following the manufacturer's protocol. Briefly, 0.5 × 10^6^ (MDA-Bone-Un), 1 × 10^6^ cells (SUM-159PT), and 0.3 × 10^6^ cells (MDA-Bone-Un CC and KO) were employed for each of the unstained gating control, DEAB (N,N-diethylaminobenzaldehyde) negative control and the test sample. Following the addition of the reagents, the cells were incubated at 37°C for 45 min. Subsequently, the cells were centrifuged, resuspended in ice-cold assay buffer and isolated based on the ALDH activity (conversion and retention of fluorescent BAA end product inside the cells) through Fluorescence Activated Cell Sorting (FACS).

### Isolation of BCSCs Based on Cell Surface Expression of CD44^+^/CD24^−^

A single cell suspension of cells cultured under low attachment conditions on poly-HEMA plates was produced by trituration and/or trypsinization. These cells were incubated at 37°C for 2 h to allow for the recovery of cell surface receptors. Cell surface CD44 and CD24 antigens were stained by incubating with FITC-CD44 (BD Biosciences, Cat.#−555478) PE-Cy7-CD24 (BD Biosciences, Cat.#−561646) antibodies for 1 h on ice. Unstained cells, along with corresponding isotype antibodies (Cat. #−552868 and Cat. #−555742—BD Biosciences) served as the appropriate controls.

### Maintenance of BCSCs

The FACS–sorted ALDH^+^ BCSCs were maintained under ultra-low attachment conditions in poly-HEMA coated 96-well plates (Corning™ Ultra-Low Attachment Microplates, Cat.#−07200603) or 6-well plates (Corning™ Ultra-Low Attachment Microplates, Cat. #−07200601). DMEM/F-12 (Dulbecco's Modified Eagle's Medium/Hams F-12 50/50 Mix) (Corning, Cat. #−10-090-CM) supplemented with 500 ng/μL basic fibroblast growth factor (bFGF) (Invitrogen GIBCO, Cat. #−PHG0263), 500 ng/μL human epidermal growth factor (hEGF) (Invitrogen GIBCO, Cat. #−PHG0311L), 2% B27 (Gibco™, Cat. #−17504044) and 1% Penicillin/Streptomycin was employed to maintain the FACS-sorted BCSCs routinely.

### Mammosphere Formation Efficiency (MFE) Assay

The evaluation of MFE was performed as described previously (39). Briefly, 1 × 10^3^ ALDH^+^ BCSCs of MDA-Bone-Un or SUM-159PT origins were seeded onto 96-well ultra-low attachment plates. They were maintained in DMEM/F12 media supplemented with 500 ng/μL bFGF, 500 ng/μL hEGF, 2% B27 mixture and 1% Penicillin/Streptomycin for 7 days. The mammosphere images were obtained longitudinally by employing IncuCyte® S3 Live-Cell Analysis System (Essen BioScience, Ann Arbor, MI). On day 7, MFE was calculated by employing the formula: (Number of mammospheres formed/Total number of cells seeded) × 100. A diameter of 100 μm was used as a cut-off in the determination of the mammosphere forming ability. For the assessment of secondary MFE, the BCSCs from primary MFE were collected, centrifuged and re-seeded onto 96-well plates coated with poly 2-hydroxyethyl methacrylate (poly-HEMA). The cells were monitored, and the mammospheres were counted using IncuCyte.

### Cell Viability Assays

The induction of cell death by the small molecule inhibitor Rocaglamide A (RocA) (Sigma/Aldrich, St. Louis, MO, Cat. #—SML0656) was followed by employing Deep Red Anthraquinone 7 (DRAQ7) dye (Abcam, Cat. #—ab109202). DRAQ7 is a cell impermeable, far-red fluorescent DNA dye that stains the nuclei of dead and plasma membrane-compromised cells. Importantly, it does not enter the live and intact cells. For the cell death analysis, ALDH^+^ BCSCs were treated with the indicated drug dosage for 7 days. On day 7, DRAQ7 was added to the cells (1:2,000 dilution) and incubated overnight. Dead BCSCs were tracked by DRAQ7 fluorescence by employing the EVOS cell imaging system (ThermoFisher Scientific, Rockford, IL).

The cell viability was alternatively determined on day 7 following treatment with RocA by employing the CellTiter-Glo® luminescent cell viability kit (Promega, Cat.#—G7570**)** as per the manufacturer's instructions. This quantitative assay is a homogeneous method of determining the number of viable cells in culture based on the amount of adenosine triphosphate (ATP) present inside the cells.

Briefly, to assess the cell viability, 3 × 10^3^ cells /well (SUM-159PT, SUM Pac 200 nM, MDA-Bone-Un eIF4A CC and KO) were seeded and the cells were allowed to attach and spread overnight under adherent or non-adherent low attachment conditions. Following treatment with RocA or paclitaxel, the viability of the cells was measured after 48h using CellTiter-Glo® luminescent cell viability kit.

### Development of Paclitaxel-Resistant TNBC Cell Lines

Paclitaxel-resistant cell lines were generated by a stepwise escalation of paclitaxel dosage with a recovery period in drug-free media between successive dosages over a total period of 6 months.

### Immunoblotting

BCSCs were harvested, washed with 1X phosphate-buffered saline, pH 7.5 (PBS) and lysed with lysis buffer (50 mM Tris, pH 7.5, 100 mM NaCl, 0.1% NP-40, 0.1% Deoxycholate, 5 mM EDTA) supplemented with protease inhibitor cocktail (Sigma-Aldrich, St. Louis, MO, Cat. #—P8340-5ML), phosphatase inhibitor cocktail 2 (Sigma-Aldrich, St. Louis, MO, Cat. #—P5726) and phosphatase inhibitor cocktail 3 (Sigma-Aldrich, St. Louis, MO, Cat. #—P0044). The samples were separated by 10% sodium-dodecyl-sulfate polyacrylamide electrophoresis (SDS-PAGE), transferred onto nitrocellulose membrane overnight and incubated with primary antibodies. The appropriate secondary antibodies conjugated to horse-radish peroxidase (HRP) were then added. The proteins with bound HRP were detected by employing an enhanced chemiluminescence-based kit (Amersham™ ECL™ Prime, GE Healthcare Life Sciences, Pittsburgh, PA, Cat. #—RPN2232).

Primary antibodies that were used were from Cell Signaling and Technology (unless otherwise indicated): SOX2 (Cat. #−3579), OCT4(Cat. #−2750), NANOG (Cat. #−4903) CD44 (Cat. #−5640), ALDH1A1 (Cat. #−54135), β-actin (Cat.#−4970S) ROCK1 (Cat. #−4035), Survivin (Cat. #−2808), Cyclin D1 (Cat. #−2922), Cyclin D3 (Cat. #−2936), eIF4A (Cat. #−2013), ABCG2 (Cat. #4477), ABCB1 (Cat. #13342), Cleaved caspase-3 (Cat. #−9664), Snail (Cat. #−C15D3), β-tubulin D66 (Sigma/Aldrich, St. Louis, MO, Cat. #−T0198), ABCC1 (Novus Biologicals, Cat. # IU2H10), and ABCG2 (Novus Biologicals,Cat. #−3G8), E-cadherin (BD Biosciences, Cat. #−610181). The ABC transporter antibodies from Novus Biologicals and Cell Signaling and Technology were used interchangeably.

The secondary antibodies were: Goat anti-Mouse IgG (H+L) Superclonal™ Secondary Ab conjugated to HRP (Thermo Scientific, Rockford, IL, Cat. #−A28177) or Goat anti-Rabbit IgG (H+L) SuperclonalTM Secondary Ab conjugated to HRP (Thermo Scientific, Rockford, IL, Cat. #−A27036).

### Genetic Ablation of eIF4A1

For generation of CRISPR/Cas9-mediated eIF4A1 knockout (KO), a set of CRISPR/Cas9 plasmids (Santa Cruz, Dallas, TX, Cat. #—sc-402623) were transfected into therapy-naïve and paclitaxel-resistant tumor cells using UltraCruz reagent (Santa Cruz, Dallas, TX, Cat. #—sc-395739) following the manufacturer's instructions. After 24 h, the supernatant was removed and replaced with regular media and cells were further cultured for a total of 72 h. eIF4A1 KO cells were sorted out based on the expression of green fluorescent protein (GFP). Single KO cells were isolated by limiting dilution in 96-well plates. The KO was verified by immunoblotting for eIF4A. Non-targeting CRISPR/Cas9 plasmids were employed to obtain the CRISPR-control cells (Santa Cruz, Dallas, TX, Cat. #—sc-418922).

### Statistical Analysis

All statistical analyses were performed using GraphPad Prism software, ver. 7.0 (San Diego, CA, USA). In order to determine the statistical significance in our experiments, Students *t*-tests were performed as indicated with the “*p*” value set to < 0.05. The results were expressed as the mean ± standard error of mean (S.E.M.).

## Results

Here, we examined if eIF4A would be involved in mediating or modulating the chemoresistance in breast cancer cells and whether it could be employed as an actionable molecular target in BCSC-directed therapy.

### Protein Levels of eIF4A, Pluripotency Transcription Factors, and ABC Drug Transporters Are Upregulated Upon Longitudinal Paclitaxel Treatment

**Figure 1:**
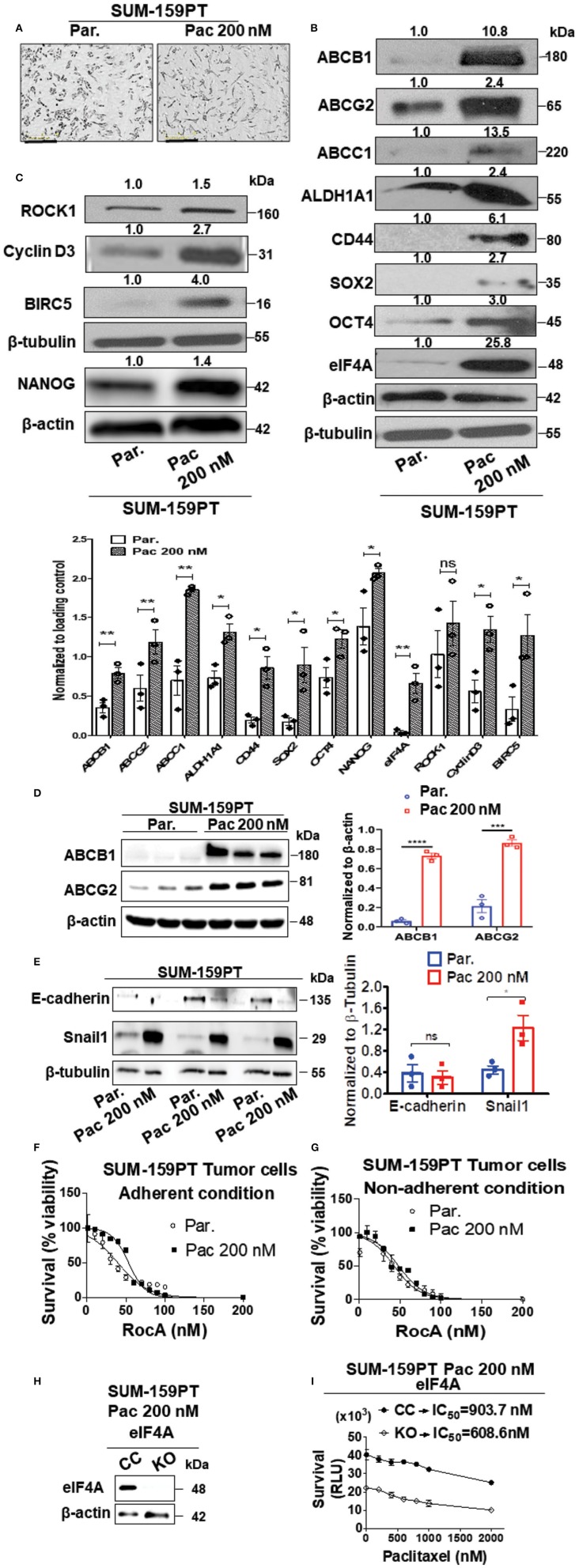
Upregulation in the levels of eIF4A, pluripotency transcription factors, and ABC transporter proteins following chronic paclitaxel treatment in SUM-159PT cells. **(A)** Micrographs depicting the morphology of the therapy naïve (par.) and the paclitaxel-resistant SUM-159PT (Pac 200) cells. Scale bar−400 μm. **(B,C)** The total proteins in the lysates from the paclitaxel-resistant SUM-159PT cells were separated by 10–12% SDS-PAGE and probed for the level of eIF4A and its downstream targets along with the key proteins involved in pluripotency, breast cancer stem-cell markers, and drug resistance by immunoblotting with specific antibodies. Fold change in the levels of proteins is indicated above the blots with the therapy naïve (par.) being normalized to 1. β-actin served as the loading control for proteins ABCC1 and OCT4 and β-tubulin served as the loading control for rest of them; (*n* = 3). The graph shows the spread of the data points along with its statistical significance, obtained by normalizing the densitometry intensity value with their corresponding loading controls. *p ≤ 0.05, **p ≤ 0.01, ***p ≤ 0.001, ****p ≤ 0.0001, ns-not significant. **(D)** The levels of key drug transporters were independently tested by immunoblotting and quantitated by densitometry and plotted (*n* = 3). **(E)** The total proteins from parental SUM-159PT cells paclitaxel-resistant SUM-159PT cells were separated by 10–12% SDS-PAGE and the expression level of E-cadherin and Snail1 (EMT markers) was analyzed and quantified. The mean expression values from *n* = 3 is represented as a bar diagram where the values are normalized to β-tubulin and indicated as “mean fold change.” **(F)** The viability of parental SUM-159PT cells and paclitaxel-resistant SUM-159PT cells was assessed following RocA treatment for 48h under adherent conditions (*n* = 3). **(G)** The viability of parental SUM-159PT cells and paclitaxel-resistant SUM-159PT cells was assessed following RocA treatment for 48 h under non-adherent, low attachment conditions (*n* = 3). **(H)** The assessment of the eIF4A knockout in paclitaxel-resistant SUM-159PT cells (*n* = 2). **(I)** The chemosensitivity of CRISPR Control and eIF4A knockout cells derived from paclitaxel-resistant SUM-159PT cells was assessed following their exposure to escalating doses of paclitaxel (*n* = 3).

Therapy resistance to the first-line chemotherapeutics is a problem in the clinic with frequent relapse in TNBC. To investigate this clinical scenario, we established a paclitaxel-resistant SUM-159PT cell line model with escalating doses of Paclitaxel (Pac) over a period of 6 months. Paclitaxel or docetaxel is an antineoplastic drug commonly employed for a wide range of cancer types. The final drug-resistant cells were routinely cultured at 200 nM paclitaxel (Pac 200). As breast cancer stem cells are known to play a vital role in chemoresistance and minimal residual disease, we examined if the breast cancer stemness would be modulated by the paclitaxel treatment (40). There were phenotypic and molecular changes that occurred after chronic exposure to paclitaxel. First, there was an alteration in the morphology in the “Pac 200” group which displayed a more elongated shape and a less tendency to group together than the control cells (Figure 1A). Second, we observed an increase in the level of stemness markers such as ALDH1A1 (2.4-fold) and CD44 (6.1-fold). Third, there was an enhanced expression of the pluripotency transcription factors such as SOX2 (2.7-fold), OCT4 (3-fold), and NANOG (1.4-fold) (Figures 1B,C). Fourth, the protein level of the ABC drug transporters namely ABCG2 or breast cancer resistance protein (BCRP), ABCB1 (P-glycoprotein) or multi-drug resistance protein (MDR1) and ABCC1 were also increased 2.4-, 10.8-, and 13.5-fold, respectively. Finally and most importantly, there was a significant increase in the expression of eIF4A (25.8-fold) (Figure 1B). This increase in the total level of eIF4A directly correlated with an enhanced expression of its downstream targets such as survivin or BIRC5 (4-fold), Cyclin D3 (2.7-fold), and ROCK1 (1.5-fold), indicative of an enzymatically active eIF4A, in the paclitaxel-resistant model (Figure 1C). The quantification of the bands from Western analysis from Figures 1B,C is graphically represented at the bottom. Most of the proteins examined in Figures 1B,C, except ROCK1 had statistically significant increase upon longitudinal exposure to 200 nM paclitaxel. ROCK1 expression showed an increasing trend albeit not statistically significant. We independently verified the enhanced expression of the drug transporters in three biological replicates with our collaborators (Figure 1D). The protein levels of ABCB1 and ABCG2 were significantly increased by 14-fold (*p* < 0.0001) and 4-fold (*p* < 0.001), respectively, similar to our findings (Figure 1B).

Several epithelial-mesenchymal transition (EMT) markers including Snail1 have been shown to play a key role in chemoresistance. To validate our model further, we examined for the expression levels of E-cadherin (a hallmark epithelial marker) and Snail1 (a mesenchymal marker) in therapy-naïve and Pac 200 SUM-159-PT cells. In paclitaxel-resistant cells, the protein level of Snail1 was significantly increased (2-fold) with a concurrent decreasing trend in the expression of E-cadherin (Figure 1E). As eIF4A showed a dramatic increase following longitudinal paclitaxel exposure, we pharmacologically targeted eIF4A with RocA in therapy-naïve and Pac 200 SUM-159PT tumor cells under both adherent and non-adherent (low attachment poly-HEMA coated) conditions. We found that RocA is effective in targeting not only in the therapy naïve but also the paclitaxel-resistant cells (Figures 1F,G). To demonstrate if eIF4A plays a role in chemoresistance, we knocked out eIF4A in Pac 200 SUM-159PT tumor cells, using the CRISPR-Cas9 approach (Figure 1H). The guide RNAs target eIF4A1 isoform specifically. Following the validation of the gene ablation of eIF4A1 by immunoblotting, we performed the viability assay to examine if genetic loss of eIF4A confers any chemosensitivity to paclitaxel in these drug-resistant cells. In particular, we examined for any gain in sensitivity following eIF4A1-KO that was derived from Pac 200 nM resistant cells by treating with escalating doses of paclitaxel up to 2 μM for 48 h (cell viability assay using CellTiterGlo kit). Importantly, the viability of the eIF4A1-KO cells decreased by 2-fold (at 0 nM paclitaxel). The CRISPR-control SUM-159PT cells had an IC_50_ of 903.7 nM and in the eIF4A1-KO cells, the IC_50_ decreased to 608.6 nM for paclitaxel (−1.5 fold change) (Figure 1I).

### Genetic Ablation of eIF4A Reduced the Expression of Stemness Transcription Factors, Drug Transporters, and Downstream Effectors of eIF4A Activity

**Figure 2:**
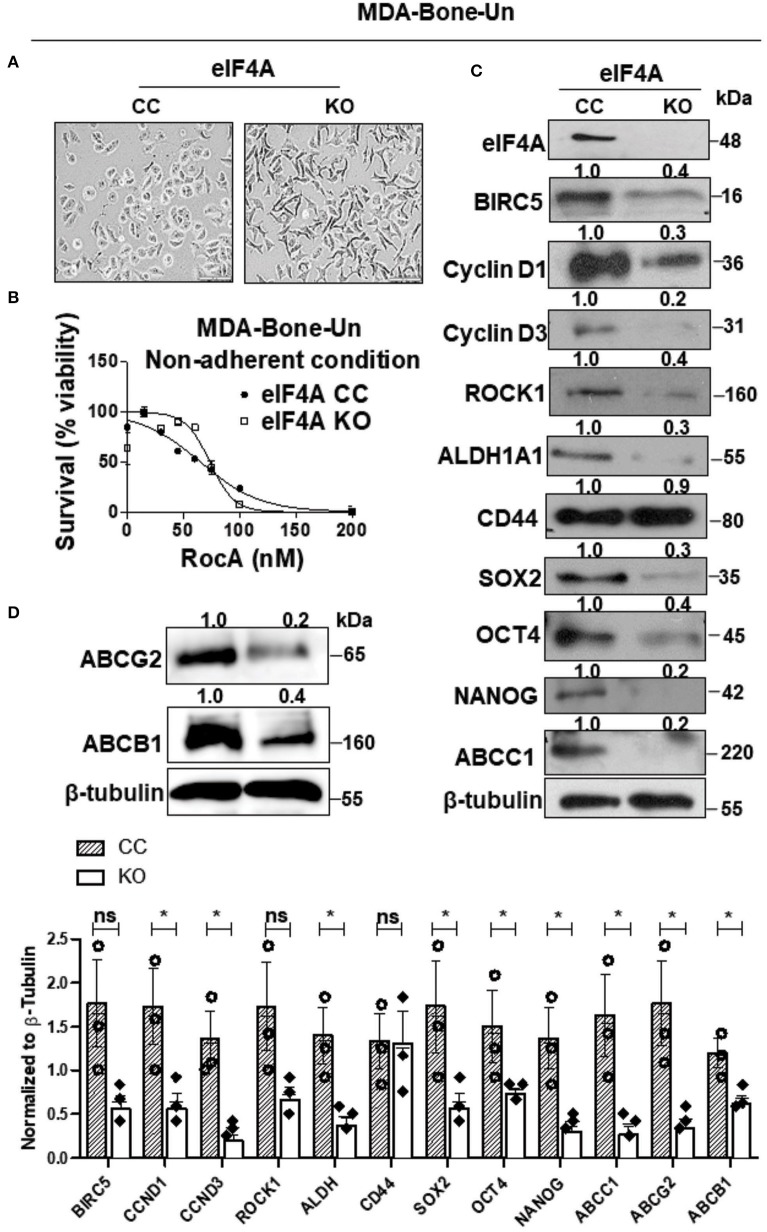
Genetic ablation of eIF4A reduces the expression of stemness transcription factors, drug transporter proteins, and downstream mediators of eIF4A signaling. **(A)** Micrographs depicting the morphology in the CRISPR-control and the eIF4A knockout MDA-Bone-Un cells. Scale bar−100 μm. **(B)** The viability of MDA-Bone-Un CRISPR Control and eIF4A knockout cells was assessed following treatment with RocA for 48 h under low attachment conditions (*n* = 3). **(C)** The total proteins in total lysates from CRISPR-control and the eIF4A knockout MDA-Bone-Un cells were separated by 10–12% SDS-PAGE and probed for expression levels of proteins downstream of eIF4A, pluripotency, BCSC markers, ABCC1 by immunoblotting with specific antibodies. β-tubulin served as the loading control (*n* = 3). Fold change in the levels of proteins is indicated above the blots with the CRISPR-Control being normalized to 1. **(D)** Immunoblot representing the differential expression of key ABC drug transporters ABCG2 and ABCB1 in the whole cell lysates from CRISPR-control and the eIF4A knockout MDA-Bone-Un cells. Fold change in the levels of proteins is indicated above the blots with the CRISPR-Control being normalized to 1 (*n* = 3). The graph following **(C,D)** shows the spread of the data along with its statistical significance. The indicated values are obtained by normalizing the densitometry intensity value with their corresponding loading controls. *p ≤ 0.05, ns-not significant.

We assessed the expression level of eIF4A in parental, lung and bone-trophic variants of MDA-MB-231 cell line. We found that eIF4A expression remained consistently similar across the cell lines (Supplementary Figure 1). We conducted further experiments with MDA-Bone-Un cells that have a higher bone-metastasizing propensity. In order to ascertain whether the dramatic increase in the expression of eIF4A in the drug-resistant “Pac 200” model has any causal relationship to the protein level of the drug transporters, eIF4A was knocked out (KO) by employing the CRISPR-Cas9 approach in therapy-naïve MDA-Bone-Un cells. The genetic loss of eIF4A induced a phenotypic change (more elongated morphology and multiple pseudopodia; Figure 2A). To check for the specificity of RocA in targeting eIF4A, we treated MDA-Bone-Un eIF4A CC and KO cells with RocA for 48 h and measured the cellular viability. We observed that the MDA-Bone-Un eIF4A KO cells were relatively insensitive up to 60 nM RocA whereas in MDA-Bone-Un eIF4A CC cells, there was a steep decrease in viability following the RocA challenge. Following further dose escalation, the drop in the viability was consistent across both cell populations (Figure 2B). This shows the specificity of RocA in targeting eIF4A in our system.

When eIF4A was genetically ablated, there was a marked decrease in the expression of the downstream targets of eIF4A such as BIRC5 (−2.5-fold), Cyclin D1 (−3.3-fold), Cyclin D3 (−5-fold), and ROCK1 (−2.5-fold) in the eIF4A-KO cells (Figure 2C). Importantly, among the two BCSC markers employed here, only the level and activity of ALDH1A1 selectively plummeted (−3.3-fold) while the expression of CD44 remained unaffected (−1.1-fold) (Figure 2C and Table 1). Interestingly, this was accompanied by a drastic reduction in the expression of the stemness transcription factors such as SOX2 (−3.3-fold), OCT4 (−2.5-fold), and NANOG (−5-fold). Finally, there was a precipitous decrease in the level of drug transporter ABCC1 (−5-fold) and marked decreases in ABCB1 (−2.5-fold) and ABCG2 (−5-fold) (Figures 2B,D).

**Table 1:** eIF4A knockout in MDA-Bone-Un cells drastically reduces ALDH activity.

**MDA-Bone-Un eIF4A**	**Biological replicate(% yield of ALDH^+^ cells)**			
	***n* = 1**	***n* = 2**	***n* = 3**	**Mean**
CC	27.5	30.8	32.4	30.23
KO	2.8	4.5	1.8	3.03

*ALDH, Aldehyde dehydrogenase; CC, CRISPR control; KO, eIF4A1 knockout*.

### Isolated ALDH^+^ Cells Are Enriched in the Expression Levels of Pluripotency Transcription Factors and Display a Higher Self-Renewal Capability

As observed in Figures 1, 2, the pharmacological treatment with paclitaxel induced an increased level of eIF4A and the breast cancer stemness. Furthermore, genetic ablation of eIF4A in TNBC cells resulted in a decrease in breast cancer stemness (reduced expression of SOX2, OCT4, and NANOG levels) mirroring the level of eIF4A. Based on these findings, we next examined whether breast cancer stemness is causally related to eIF4A by pharmacologically targeting eIF4A. RocA was employed in our study to inhibit eIF4A. In order to do so, we isolated breast cancer stem cells (BCSCs) from MDA-Bone-Un and SUM-159-PT TNBC cell lines based on the enrichment of aldehyde dehydrogenase (ALDH) activity. This was accomplished by FACS based isolation using the “Aldeflour” kit. With the DEAB inhibitor serving as the negative control, 14.6% of MDA-Bone-Un (Supplementary Figure 2A) and 3.5% of SUM-159PT (Supplementary Figure 2B) tumor cells were enriched for ALDH activity. Following isolation, the ALDH^+^ cells were maintained under low attachment conditions in poly-HEMA coated plates where the cells formed distinct mammospheres (Supplementary Figures 2A,B).

In order to evaluate whether the isolated ALDH^+^ populations were enriched for cancer stemness, we examined the expression level of the pluripotency transcription factors such as SOX2, OCT4, and NANOG along with ALDH1A1 (one of the key isoforms in the ALDH family of enzymes regulating the cancer stem cell phenotype) by immunoblotting of total lysates from ALDH^+^-BCSCs. As expected, the levels of proteins implicated in pluripotency were enhanced in BCSCs with high ALDH activity. In particular, a 2-fold increase in the expression of ALDH1A1 and SOX2 and 1.7-fold increase in OCT4 levels was observed in ALDH^+^ cells compared to ALDH^−^ cells (Supplementary Figure 3A). NANOG levels were comparably similar (data not shown) between the ALDH^+^ and ALDH^−^ populations. Next, we compared the ability to self-renew by the ALDH^−^ and the ALDH^+^ populations through the determination of the efficiency of formation of the primary and secondary mammospheres (MFE). The primary (3-fold, *p* < 0.0002) (Supplementary Figures 3Bi,C) and the secondary (3-fold, *p* < 0.0001) (Supplementary Figures 3Bii,C) MFE were significantly higher for MDA-Bone-Un ALDH^+^-BCSCs compared to their ALDH^−^ counterparts. Next, we similarly examined the cancer stemness characteristics for the ALDH^+^ cells isolated from a second TNBC cell line, SUM-159PT cells. There was a 2-fold increase in the level of ALDH1A1 protein in the BCSCs from SUM-159PT cells. The BCSCs were also enriched for SOX2 and NANOG (2.8-fold) than the ALDH^−^ cells (Supplementary Figure 3D). The primary and the secondary MFE were also significantly higher for ALDH^+^ (2-fold, *p* < 0.0006 for primary and 3-fold, *p* < 0.0001 for secondary mammospheres) than the ALDH^−^ cells (Supplementary Figures 3Ei,ii,F).

### ALDH^+^ Cells Co-express CD44 Marker

Increased ALDH activity and CD44 (CD44^hi^/CD24^low^) expression have been identified as some of the key markers for breast cancer stemness. To evaluate if the isolated ALDH^+^ cells also express CD44, we examined for its expression by FACS analysis as well as immunoblotting. FACS analysis revealed that more than 90% of the isolated ALDH^+^-BCSCs co-expressed CD44 (CD44^hi^/CD24^low^), both from MDA-Bone-Un (Supplementary Figure 4A) and SUM-159PT (Supplementary Figure 4C) cells. Further validation by immunoblotting also revealed a high co-expression of CD44 along with ALDH1A1 marker (Supplementary Figures 4B,D). Thus, we confirmed that more than 90% of were ALDH^+^ and CD44^hi^ and CD24^low^ double positives.

### Targeting of eIF4A Induced BCSC Death and Reduction in the Self-Renewal Ability of the BCSCs

**Figure 3:**
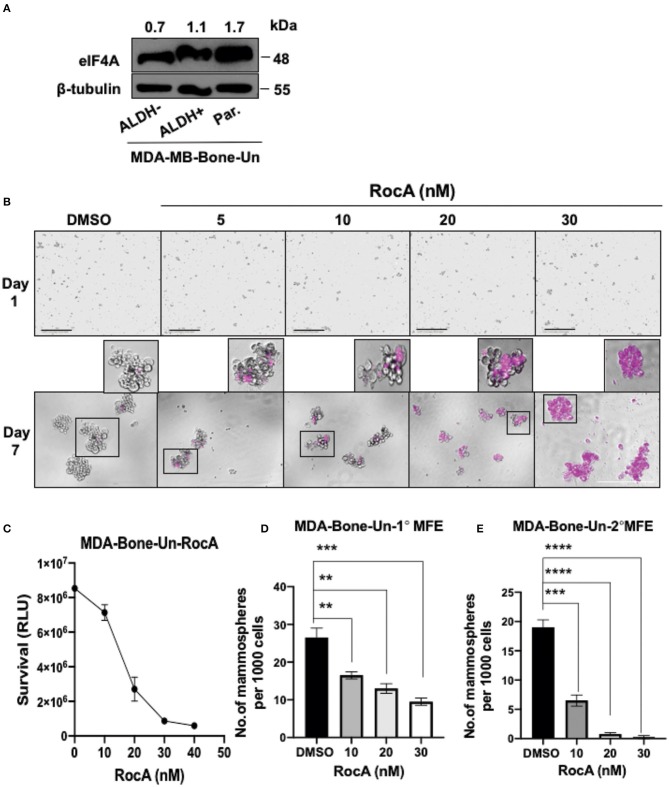
Targeting eIF4A induces cell death and reduces the self-renewal ability of BCSCs derived from MDA-Bone-Un cells. **(A)** Immunoblot showing expression levels of eIF4A between the ALDH^−^ and ALDH^+^ and the unsorted, parental MDA-Bone-Un cell populations. **(B)** RocA-induced cell death in the BCSCs was assessed by DRAQ7 staining and images were captured by light microscopy. DRAQ7 staining is carried out on day 7 following the treatment; Scale bar−400 μm. **(C)** The viability of cells following RocA treatment was measured by employing CellTiter-Glo assay. **(D,E)** Represents the reduction in the primary and the secondary MFE following treatment. Data are presented as Mean ± S.E.M. (*n* = 3). **p ≤ 0.01, ***p ≤ 0.001, ****p ≤ 0.0001.

As eIF4A expression dramatically increased in drug-resistant tumor cells, we evaluated if eIF4A could be a potential drug target in BCSCs. We initially examined whether eIF4A is expressed in our double positive BCSCs (ALDH^+^ and CD44^+^), ALDH^−^ cells and the non-sorted, parental tumor population from MDA-Bone-Un cells. We found that eIF4A was consistently and uniformly expressed in parental and bulk tumor cells (ALDH^−^ cells) and BCSCs from the MDA-Bone-Un tumor cell line (Figure 3A). As the target eIF4A was expressed in the isolated BCSCs, RocA was employed to inhibit eIF4A. The mammospheres that were routinely cultured in 6-well dishes were uniformly seeded onto a 96-well plate under low attachment conditions and termed as “Day 1” for DMSO control and RocA treatment (5–30 nM; Figure 3B, top panel). To assess the impact of RocA on BCSCs, the BCSCs were continually incubated with various concentrations of RocA for 7 days. The effects of RocA on the survival and the self-renewal abilities of the BCSCs were evaluated at the end of the 7th day. The cell death was assessed by DRAQ7 assay. It is clearly evident that DRAQ7 failed to stain the control BCSCs while RocA treated groups demonstrated intense DRAQ7 staining depicting dead BSCSs (pseudo-colored purple) even at 30 nM of RocA (Figure 3B, bottom panel). At higher concentrations of RocA (60–100 nM), the size of the mammospheres were reduced (data not shown) and more fragmented purple cellular debris was evident. Next, the cell survival (or death) was quantified by employing an alternate approach that examined the level of cellular ATP as a measure of viability using the “CellTiter-Glo” assay. The half maximal inhibitory concentration (IC_50_) of RocA was found to be 15 and at 30 nM nearly all BCSCs were wiped out based on the ATP level reflecting the mitochondrial activity (Figure 3C). This correlated well with the DRAQ7 assay where almost all BCSCs appear to be stained by DRAQ7 (Figure 3B, bottom panel). Finally, the ability of the BCSCs to self-renew was assessed by the efficiency of the primary and secondary mammosphere formation (MFE). The control group formed the mammospheres efficiently under low attachment conditions (30 primary mammospheres/1,000 cells). However, in the RocA-treated group, the self-renewal capability of BCSCs was significantly impaired with nearly a 50% reduction of the primary mammospheres even at 10 nM RocA concentration (*p* < 0.0001; Figure 3D). The secondary MFE was severely impacted at 10 nM RocA with a significant reduction in the mammospheres (*p* < 0.0001; Figure 3E).

**Figure 4:**
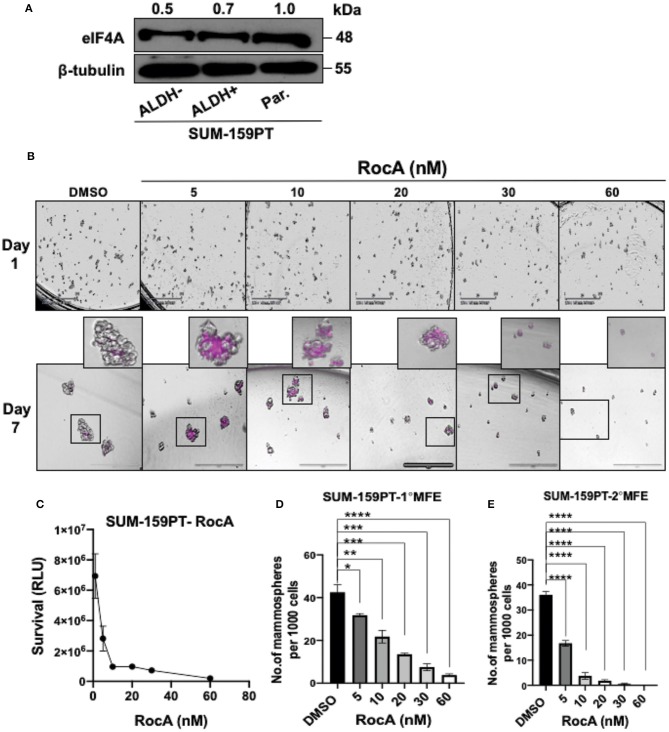
Targeting eIF4A induces cell-death and reduces the self-renewal ability of BCSCs derived from SUM-159PT cells. **(A)** eIF4A levels were analyzed between the ALDH^+^ and ALDH^−^ and the unsorted, parental SUM-159PT cells by immunoblotting (*n* = 3). **(B)** Cell death was visualized via DRAQ7 staining following RocA treatment on day 7. Scale bar−400 μm (*n* = 3). **(C)** The cell viability analysis using CellTiter-Glo assay in SUM-159PT cells following RocA treatment (*n* = 3). **(D,E)** The self-renewal ability was measured by the primary and the secondary MFE following RocA treatment (*n* = 3). Data are presented as Mean ± S.E.M. *p ≤ 0.05, **p ≤ 0.01, ***p ≤ 0.001, ****p ≤ 0.0001.

Next, we targeted eIF4A pharmacologically in the BCSCs derived from a second cell line SUM-159-PT. eIF4A was uniformly expressed in the parental, bulk tumor cells (ALDH^−^ cells) and BCSCs (Figure 4A). As the BCSCs from SUM-159-PT cells were found to be more sensitive to RocA, the efficacy of RocA in inducing cell death was examined from 5 to 60 nM (Figure 4B). More than 50% of the BCSCs were intensely stained with DRAQ7 at 5 nM of RocA. At higher concentrations of RocA, a similar trend was observed i.e., intense staining of 50% of the cells but also additional milder staining of more cells was noted. Next, we studied the viability by the CellTiter-Glo' assay (Figure 4C). When BCSCs were incubated with 5 nM RocA, more than 50% of cells were dead as opposed to 15 nM of RocA for BCSCs derived from MDA-Bone-Un for a similar outcome. With regard to the formation of the primary mammospheres, there was a 50% reduction at 10 nM RocA which further plummeted at higher concentrations of RocA (Figure 4D). The decrease in secondary MFE was more drastic (Figure 4E).

### Pharmacological Targeting of eIF4A in MDA-Bone-Un BCSCs Affects the Expression of Pluripotency Transcription Factors, ALDH1A1 and Induces Apoptotic Cell Death

**Figure 5:**
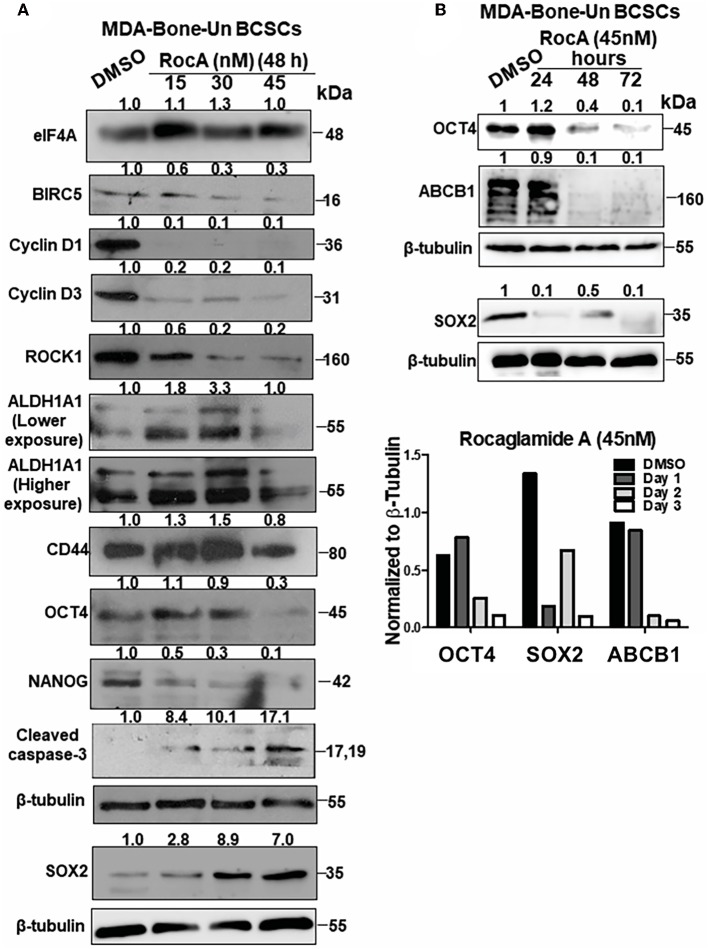
Targeting of eIF4A in BCSCs derived from MDA-Bone-Un cells affects the pluripotency transcription factors, ALDH1A1, drug transporter ABCB1 and induced apoptotic cell death. **(A)** Representative immunoblot showing the dose-dependent effects of RocA on the levels of eIF4A, molecular targets downstream of eIF4A, pluripotency transcription factors and BCSC markers ALDH1A1 and CD44 (*n* = 3). **(B)** The given immunoblot is obtained following RocA treatment of MDA-Bone-Un derived BCSCs at 45 nM for a period of 72 h. The lysates from three biological replicates were pooled and assessed for the expression of SOX2, OCT4, and ABCB1. Fold change in the levels of proteins is indicated above the blots with DMSO control being normalized to 1. The graph following the immunoblot represents the densitometry values normalized to their respective loading controls. The graph corresponding to **(A)** shows the spread of the data along with its statistical significance. The indicated values are obtained by normalizing the densitometry intensity value with their corresponding loading controls. *p ≤ 0.05, **p ≤ 0.01, ***p ≤ 0.001 ns-not significant. The graph corresponding to **(B)** shows the trend of SOX2, OCT4, and ABCB1 from 3 biological replicates pooled and analyzed.

**Figure 6:**
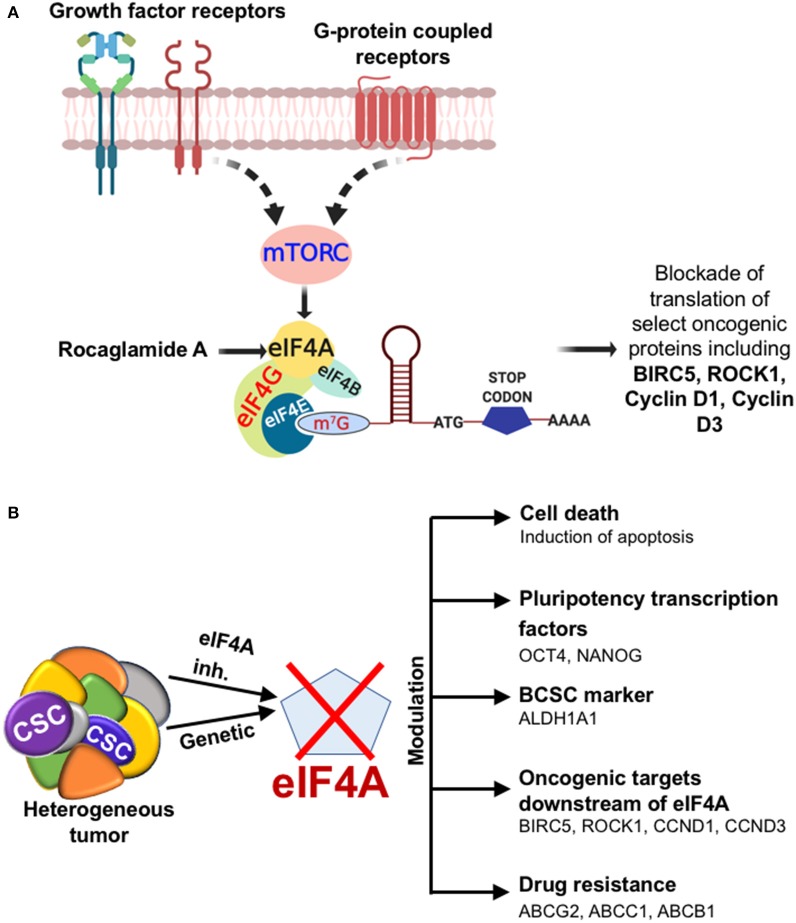
Diagrammatic illustration of the effects of targeting eIF4A. **(A)** The cartoon depicting the outcome when eIF4A is targeted in the breast tumor. The cartoon depicts the growth factor receptor and GPCR pathways impinging on the key signaling node eIF4A through a common upstream effector mTORC in breast cancer. The significance of targeting eIF4A pharmacologically is brought about by the selective blockade of oncogenic targets downstream of eIF4A. **(B)** Depiction of the impact of eIF4A in the heterogeneous breast tumor and the modulation of breast cancer stemness, levels of key oncoproteins, and drug transporters.

To assess the mechanism of cell death in RocA-treated BCSCs from MDA-Bone-Un, BCSCs were treated with 15, 30, and 45 nM of RocA for 48 h (short-term exposure as opposed to the chronic paclitaxel treatment). We initially assessed whether RocA had hit the target eIF4A by immunoblotting for the expression of the downstream effectors (BIRC5, Cyclin D1, Cyclin D3, and ROCK1) of eIF4A activity. Following treatment, we observed a dramatic reduction in the expression of BIRC5, Cyclin D1, Cyclin D3, and ROCK1 (Figure 5A). RocA treatment did not affect the total protein level of eIF4A. Having confirmed that the activity of eIF4A was compromised following RocA treatment, we next tested whether the level of the pluripotency transcription factors would be modulated. Of the three transcription factors examined, the expression of NANOG was significantly reduced (a 2- and 10-fold reduction in NANOG level at 15 and 45 nM RocA, respectively). OCT4 was reduced by 3.3-fold at 45 nM RocA level while the SOX2 level remained elevated above the basal level at all concentrations of RocA for 48 h. The levels of the BCSC markers ALDH1A1 and CD44 were not affected at 48 h. Importantly, there was an induction of cleaved caspase-3 (up to 17.1-fold increase) when treated with RocA. This clearly indicates that cell death in BCSCs occur through apoptosis following the RocA treatment (Figure 5). Next, we fixed the concentration of RocA at 45 nM and examined the levels of OCT4, SOX2, and ABCB1 over 72 h. The 3 biological replicates were pooled and analyzed. OCT4 decreased by 2.5-fold at 48 h and 10-fold at 72 h. SOX2 level decreased by 10-fold in 24 h and increased back by 2-fold in 48 h. At 72 h, degradation of SOX2 was observed though the loading control β-tubulin band was intact, indicating live cells. Importantly, the ABC transporter ABCB1 was dramatically reduced by 10-fold at 48 and 72h. The normalized ratiometric quantitation was shown in the bottom graph (Figure 5B). A general schema of various molecular signaling pathways impinging on eIF4A and the impact of RocA on oncogenic targets in BCSCs and the resultant outcome are presented pictorially (Figures 6A,B).

## Discussion

Oncogenic protein synthesis is a tightly regulated process, with translation initiation being the rate limiting step governed by the eIF4F complex. The eIF4F complex consists of three core subunits: eIF4E, the cap binding subunit; eIF4A, an RNA helicase, and eIF4G1, a large scaffolding protein. The auxiliary protein eIF4B enhances the activity of eIF4A. The core component eIF4A has been documented to promote the translation of oncogenic mRNAs with stem-loop structure (SLS) in their 5′-untranslated region (5′-UTR) in cancer (41). The resultant oncoproteins constitute the rewired pro-tumor proteome that is vital for breast cancer cell survival, tumor progression, local invasion and metastasis (42–52).

The role of the eIF4F complex is increasingly evident in all types of solid tumors and hematological malignancies (53). Chemoresistance and therapy failure is a frequent clinical issue in cancer patients. Interestingly, the eIF4F complex is reported to form a nexus of drug resistance to antineoplastic therapies in melanoma (54). Disrupting the eIF4F complex formation by targeting eIF4A and other core subunits has been shown to synergize with BRAF inhibitors. Thus, targeting of the eIF4F complex has been implicated in alleviating the drug resistance or sensitizing cancer cells to other forms of chemotherapy (49, 54–56). The role of eIF4A in breast cancer has not been extensively studied. Suppression of eIF4A activity has been suggested to affect maintenance and progression of breast cancer (57). A large scale tissue microarray study involving about 4,000 patients documented that the expression of eIF4A and eIF4B can predict the clinical outcome in estrogen receptor-negative (ER-) breast cancer and was statistically independent from other known prognostic factors (58). This highlights the fact that eIF4A is a clinically relevant target in combating chemoresistance and therapy failure in metastatic breast cancer (59).

We recently reported that CXCR4-LASP1-eIF4A axis promotes translation of oncogenic proteins such as survivin, cyclin D1, MDM2, and ROCK1 in TNBC (34). Here, we report for the first time that there was an upregulation in the level of eIF4A following chronic paclitaxel treatment in SUM-159PT cells. This change in eIF4A level positively correlated with the protein level of its downstream targets such as survivin and cyclin D1 reflecting an increase in enzymatically active eIF4A in the paclitaxel-resistant TNBC cells. This was accompanied by an increase in breast cancer stemness (SOX2, OCT4, and NANOG levels) and significant increase in the levels of key drug transporters (ABCG2, ABCB1, and ABCC1). The associated morphological changes appeared to be pro-migratory in nature. This is similar to a clinical situation where the patients have been subjected to multiple clinical trials or receiving multiple drugs over a period of time. On the contrary, the CRISPR-Cas9-mediated genetic ablation of eIF4A in MDA-Bone-Un cells (MDA-MB-231 cell line that had undergone mesenchymal-epithelial transition at the metastatic site as this was re-isolated from mouse bone metastatic lesions) resulted in altered morphology. The eIF4A-KO led to a severe reduction in the expression of its target genes validating the genetic loss of eIF4A. The expression of the pluripotency transcription factors SOX2, OCT4, and NANOG was impaired which will reduce the stemness and render them more susceptible to therapy. Interestingly, the ALDH1A1 level decreased dramatically and would render these cells susceptible to chemotherapeutic drugs as a result of impairment of the ability to detoxify drugs. The selective decrease in the level of ALDH1A1 was interesting as the level of the other major BCSC marker CD44 was unaltered. This may explain their susceptibility to RocA and also sensitization to other therapeutic agents as the upregulation of the ALDH activity is correlated with high tumorigenic potential, self-renewal capability and the generated tumors from the minimal residual disease recapitulate the heterogeneity of the parental tumor. Importantly, in a series of 577 breast cancer specimens, the ALDH1 detected by immunohistochemical staining correlated with poor prognosis (60). Furthermore, the key drug transporter levels (ABCG2 and ABCC1) were greatly reduced. This is an interesting finding as targeting of eIF4A have been reported to sensitize the tumor cells to tumor necrosis factor-related apoptosis-inducing ligand (TRAIL)-induced apoptosis or break TRAIL resistance in tumor cells (61–66).

The uniform presence of eIF4A in parental, BCSCs and ALDH^−^ cells (bulk tumor cells) indicate that targeting of eIF4A would eliminate both bulk tumor cells and BCSCs simultaneously. Blocking the activity of eIF4A in BCSCs through RocA treatment, curtailed the self-renewal capability as indicated by the significant decrease in primary and secondary MFE. NANOG level was severely reduced while OCT4 level plummeted at 45 nM RocA. The variability in levels of SOX2 was observed in the first 48 h of RocA treatment but by 72 h even SOX2 was observed as degraded products. The regulatory pathways downstream of NANOG through its direct or indirect activity regulates several aspects of tumorigenesis, self-renewal, epithelial-mesenchymal transition (EMT), cell motility, immune evasion, and drug resistance (19). So the decrease in level of NANOG would have a significant bearing on the clinical outcome. On the contrary, ectopic expression of OCT4 and NANOG in lung adenocarcinoma induced cancer stemness and EMT (67). The eukaryotic translation initiation factor eIF4G is known to function as a scaffold protein and activate eIF4A. When 4EGI-1, an inhibitor of the interaction between eIF4E and eIF4G and hence inhibition of eIF4A activation, was applied to BCSCs it effectively inhibited their proliferation. The resultant protein profile of the 4EGI-1 treated BCSCs was very similar to our findings in that NANOG, OCT4 levels were downregulated (68). On the contrary, some variability in SOX2 response was observed as SOX2 might probably compensate for the loss of NANOG and OCT4. Interestingly, the expression of OCT4 but not SOX2 correlated with poor prognosis in surgical TNBC patients (22). SOX2 has been shown to transactivate the Cyclin D1 promoter which would facilitate proliferation and clonogenicity working in conjunction with cyclin-dependent kinases 4/6 (69, 70). However, in our study there was a paradoxical increase in SOX2 expression but with drastic reduction in the level of cyclin D1 indicating that this is due to the blockade of eIF4A by RocA. DRAQ7 and viability assays indicated cell death in BCSCs following RocA treatment. Induction of cleaved caspase-3 indicated that the BCSCs are primarily undergoing cell death through an apoptotic process upon exposure to RocA. Importantly, treatment of BCSCs with 45 nM RocA showed a dramatic decrease in ABCB1 by 48 and 72 h indicating a possible reversal of chemoresistance in BCSCs. Later time points demonstrated that the BCSCs were dying as per our viability assay and furthermore we observed a decrease in proteins products of housekeeping genes like β-tubulin.

Overall, our study shoes that eIF4A would be a feasible target against breast cancer stemness. It also has the advantage of clearing out both bulk tumor cells and BCSCs simultaneously with a single drug. Alternatively, the small molecule inhibitors against eIF4A could be synergistically combined with the first-line of therapy or other targeted inhibition modalities including immunotherapy.

## Data Availability Statement

All datasets generated for this study are included in the article/Supplementary Material.

## Author Contributions

SS: co-design and execution all of the experiments, analysis, plotting and organization of the data, editing, and co-writing of the paper. MR: isolation of the breast cancer stem cells based on ALDH activity initially in the project. DB-T: contributed Figure 1D and editing of the paper. CH: immunoblotting of selected proteins in drug-resistance experiments, editing of the paper, and intellectual discussions. BS: intellectual discussions. AMCT: intellectual discussions and polishing of the Abstract of the paper. AKT: intellectual discussions, some contribution to the experimental designs, and editing of the paper. DR: co-design of all of the experiments, critical analysis of the data and feedback, intellectual discussions, editing, and co-writing of the paper.

### Conflict of Interest

The authors declare that the research was conducted in the absence of any commercial or financial relationships that could be construed as a potential conflict of interest.

## Supplementary Material

Supplementary Figure 1 eIF4A expression levels remain consistent across the different metastatic variants of MDA-MB-231 cells. eIF4A expression was assessed across the different metastatic variants of MDA-MB-231 cells. MDA-Bone-Un and MDA-MB-1833 metastasize to the bone whereas MDA-MB-4175 metastasizes to the lungs (*n* = 4).

Supplementary Figure 2 Isolation of ALDH^+^ cells by flow cytometry based on enrichment of intracellular ALDH activity in two TNBC cell lines. Aldefluor assay depicting the isolation of ALDH^+^ cell population obtained after gating with the negative control DEAB and analysis by flow cytometry in **(A)** MDA-Bone-Un cells and **(B)** SUM-159PT along with the corresponding representative micrographs of ALDH^−^ cells cultured in monolayer and ALDH^+^ population grown under low attachment poly-HEMA coated plates in the bottom panel. Scale bar−50 μm for the ALDH^−^ and ALDH^+^ populations from MDA-Bone-Un and SUM-159PT cells.

Supplementary Figure 3 Isolated ALDH^+^ cells are enriched in the expression of pluripotency transcription factors and display a higher ability for self-renewal. **(A)** Immunoblot analysis showing the protein levels of ALDH1A1, SOX2 and OCT4 in the isolated ALDH^+^ population vs. the ALDH^−^ population in MDA-Bone-Un cells. **(B)** (i, ii) ALDH^−^ and ALDH^+^ population from MDA-Bone-Un were compared for their self-renewal potential by assessment of primary and secondary mammosphere formation efficiency (*n* = 3). **(C)** Pictorial representation of the primary and secondary mammospheres formed by the ALDH^−^ and ALDH^+^ population isolated from MDA-Bone-Un. Scale bar- Primary mammospheres− 800 μm, Secondary mammospheres−800 μm. **(D)** Immunoblot showing the levels of expression for ALDH1A1, SOX2, NANOG in the sorted ALDH^+^ population vs. its ALDH^−^ counterpart in SUM-159PT cells. **(E)** (i, ii) SUM-159PT derived ALDH^−^ and ALDH^+^ population were compared for their self-renewal potential by assessment of primary and secondary mammosphere formation efficiency (*n* = 3). **(F)** Represents the primary and secondary mammospheres formed by the ALDH^−^ and ALDH^+^ population sorted from SUM-159PT. Scale bar- primary and secondary mammospheres−800 μm. Data are presented as Mean ± S.E.M.

Supplementary Figure 4 ALDH^+^ cells co-express CD44. The ALDH^+^ BCSCs co-express CD44, the cell surface BCSC marker as assessed by flow cytometric analysis in **(A,C)** and confirmed by immunoblotting for CD44 in **(B,D)** in MDA-Bone-Un and SUM-159PT cells respectively (*n* = 3).
